# A novel radiation protection method for miniaturized MIMO mobile terminal antenna design based on metamaterials

**DOI:** 10.1371/journal.pone.0323299

**Published:** 2025-05-07

**Authors:** Wen-Ying Zhou, Yu-Xin Li, Wei Li, Mai Lu, Jin-Jing Xu

**Affiliations:** 1 Key Laboratory of Opto-Electronic Technology and Intelligent Control of Ministry of Education, Lanzhou Jiaotong University, Lanzhou, China; 2 Zhiyuan Laboratory, Hangzhou, China; Universiti Brunei Darussalam, BRUNEI DARUSSALAM

## Abstract

In this article, we propose an innovative approach to reduce radiation dose absorption inside human head tissues by shrinking the multiple-input multiple-output (MIMO) terminal geometric area. Initially, we employ COMSOL software to design a MIMO mobile terminal antenna that meets 2G, 3G, 4G, and 5G communication requirements. Through adding the decoupling unit, its geometric area reduces from 58 × 120 mm² to 44 × 80 mm², and its simulations and measurements indicate that the miniaturized MIMO mobile terminal antenna exhibits good radiation performance. Subsequently, we construct a head model based on standard anatomical features, including the scalp, skull, cerebrum, cerebellum, and brainstem. A comparative analysis of the specific absorption rate (SAR) across various cranial tissues, conducted before and after the antenna’s miniaturization, reveals significant reductions: maximum decreases of 85.51% in the scalp, 85.62% in the skull, 89.02% in the cerebrum, 93.04% in the cerebellum, and 88.02% in the brainstem. These findings suggest a significant decrease in the risk of electromagnetic exposure to human subjects by miniaturization. The miniaturization of the MIMO mobile terminal antenna could effectively mitigate the absorption of radiation by head tissues, thereby presenting a novel strategy for electromagnetic radiation protection.

## 1 Introduction

As wireless communication technology becomes increasingly embedded in everyday life, it significantly contributes to the complexity and variability of environmental electromagnetic fields [1], primarily due to the multifrequency and pervasive utilization of wireless devices [2,3]. Simultaneously, the widespread implementation of radio frequency (RF) radiation raises considerable concerns about potential health risks.

MIMO technology represents a major advance in modern wireless communications, with multiple antenna elements working simultaneously to provide higher data transfer rates [4–7]. However, antenna design must balance portability requirements—such as miniaturization [8,9], thinness [10], lightweight [11] and lower SAR [12] with optimal radiation performance. In antenna arrays, electromagnetic interactions between elements (known as mutual coupling effects) can substantially alter the radiation pattern and reduce efficiency, particularly when elements are closely spaced [13]. This phenomenon results in energy transfer and diminished radiation capacity. To address these challenges, researchers have developed advanced solutions: controlling electromagnetic wave propagation via metamaterials, balancing coupling currents with neutralizing lines, redirecting coupling energy through parasitic elements, and optimizing current distribution using defected ground structures (DGS). These technologies not only mitigate mutual coupling but also enable multi-antenna integration in compact devices, thereby enhancing radiation performance while maintaining portability. For instance, Ref. [14] introduced an 8-port dual MIMO system that employs a curved structure to integrate a 4-MIMO configuration onto the chassis, facilitating antenna miniaturization. In [15], an inverted L-shaped partial grounding technique was proposed to enhance isolation between antenna elements. Ref. [16] discussed the miniaturization of MIMO antennas through the incorporation of a dielectric substrate layer positioned between the metal patch and the ground. Additionally, Ref. [17] and [18] explored the use of artificial electromagnetic metamaterials, which not only enhance structural adaptability but also preserve favorable radiation characteristics. Ref. [19] presented an innovative mesh structure embedded within the radiation plane and the DGS, which aids in the coupling of currents generated by the excitation antenna while utilizing the DGS for targeted suppression, thus improving isolation performance. Ref. [20] described a balanced slot design characterized by symmetrically distributed currents of similar amplitude but opposite direction, resulting in a low SAR current distribution. This balanced mode significantly mitigates the electric field component of radiation that penetrates the human body. Furthermore, Ref. [21] employed a metasurface array as the backing element of the antenna to diminish back radiation and enhance the radiation performance in proximity to the human body. Consequently, the metamaterials decoupling structure presented in this study can minimize the distance between antenna elements while ensuring a high degree of isolation. This advancement plays a significant role in the miniaturization of MIMO mobile terminal antennas. However, the recent studies on smartphone MIMO antennas revealed persistent gaps. Ref. [22] achieved 95% efficiency and 11dBi gain in 4–10 GHz using DGS and metamaterials but lacks mm-wave compatibility and underperforms in mutual coupling (MC) suppression for multi-port systems. Ref. [23] targeted 28/38 GHz with decoupling structures (DS), reducing MC to -60 dB, yet isolation remains inferior to EBG-based solutions. Ref. [24] employed electromagnetic bandgap (EBG) for superior MC suppression but neglects cost-effectiveness and multi-band integration. Collectively, these efforts failed to harmonize ultra-wideband coverage (Sub-6 GHz to mm-wave), extreme miniaturization, and sub-30 dB MC across diverse bands, while inadequately addressing scalable fabrication and cost-performance trade-offs. Bridging these gaps necessitated hybrid techniques and low-cost substrates without compromising SAR compliance.

Numerous studies in the field of bioelectromagnetics utilize epidemiological methodologies [25] and numerical electromagnetics [26] to evaluate the safety of electromagnetic exposure during communication activities [27]. For instance, alterations in cortical excitability may be associated with sleep disturbances, with heightened cortical excitability and efficiency potentially persisting for several minutes following exposure [28]. The RF electromagnetic radiation emitted by wireless devices was demonstrated to affect the expression and functionality of the transcription factor C/EBPβ in oligodendrocytes within the cerebrum [29]. Additionally, electronic devices such as computers and mobile phones pose significant risks to cellular growth [30]. Through the application of computational electromagnetics, researchers were able to quantify the radiation dose absorbed by human tissues. A significant body of research concentrated on assessing the SAR in the human head during the utilization of mobile terminals [31–33]. Furthermore, wearable devices were found to exert a more pronounced radiation impact on human tissues due to their proximity to the skin. Notably, the maximum SAR recorded significantly exceeded the threshold established by occupational safety and health standards [34]. One study indicated that the SAR for a child model exposed to the electromagnetic radiation environment of mobile phones was 2.5 times greater than the maximum allowable value defined by regulatory standards [35]. Consequently, the evaluation of the effects of electromagnetic radiation (EMR) on human tissues, in conjunction with epidemiological data, as well as the development of effective radiation protection strategies, represent critical areas of research.

In this article, an innovative radiation protection approach is proposed, by using the miniaturized MIMO mobile terminal antenna to reduce the SAR on the human head. We adapt metamaterials units to enhance the isolation between antenna units and realize the miniaturization of the MIMO mobile terminal antenna. The operational frequency of this antenna encompasses the bands utilized in contemporary wireless communication systems, specifically 2G (1.875 ~ 1.925 GHz), 3G (2.085 ~ 2.125 GHz), 4G (2.55 ~ 2.64 GHz), and 5G (3.41 ~ 3.55 GHz). Simulations and measurements indicate that the proposed miniaturized antenna meets the communication requirements, including multi-band coverage and high gain. Furthermore, simulations of the radiation dose absorbed by various tissues within the human head are conducted. A comparative analysis of the SAR distribution in the head tissues before and after the miniaturization of the antenna reveals a significant reduction in the impact on the human head, even with increases in operational frequency and input power. This finding offers a promising strategy for enhancing RF electromagnetic protection in mobile terminal devices.

## 2 Miniaturized MIMO mobile terminal antenna design

### 2.1 MIMO mobile terminal antenna design and its miniaturization

**Fig 1:**
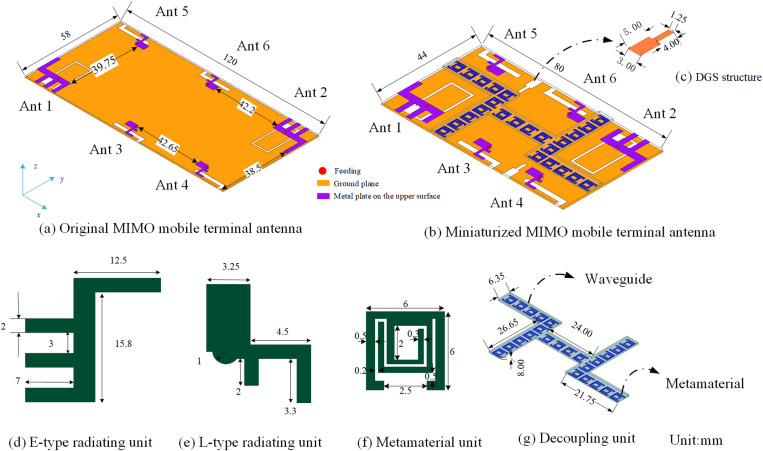
Geometry and structure of the MIMO mobile terminal antenna.

To facilitate seamless handover and ensure stable communication across diverse mobile communication network environments, we develop a MIMO mobile terminal antenna. The geometric configuration of this antenna is depicted in Fig 1a. The dimensions of the FR4 dielectric substrate are 58 × 120 × 0.8 mm³, with a relative permittivity of 4.4. Six antenna units are integrated on the upper surface of the substrate. Fig 1d provides a detailed depiction of the E-type radiating units, designated as Ant1 and Ant2, which operate within the frequency bandwidths of 1.875 ~ 1.925 GHz and 3.41 ~ 3.55 GHz. Additionally, Fig 1e illustrates the geometry of the L-type radiating units, which include Ant3, Ant4, Ant5, and Ant6. These units function within the frequency ranges of 2.085 ~ 2.125 GHz and 2.55 ~ 2.64 GHz. These bands meet 2G, 3G, 4G, and 5G communication requirements.

The miniaturized MIMO terminal antenna is illustrated in Fig 1b, with the dimensions of the FR4 dielectric substrate reduced to 44 × 80 × 0.8 mm³. To overcome the coupling effects between the antenna units, we propose the decoupling units, which combine the metamaterials units and waveguides, and a DGS structure in the ground plane. In the proposed decoupling unit, the waveguide is covered on the metamaterials to reduce the coupling between the 3.5 GHz antenna units, as shown in Fig 1g.

By etching specific patterns onto the ground plane to create a distinct defect structure, the DGS alters the distribution of shielding currents on the ground plane. This modification results in corresponding changes to the equivalent inductance and capacitance of the transmission line, thereby producing band-stop characteristics within a designated frequency range to facilitate decoupling. Our design aims to minimize the coupling effect in the 2.1 GHz band utilizing the DGS structure, as shown in Fig 1c, which significantly enhances the performance and stability of wireless communication devices.

### 2.2 Toroidal metamaterials

**Fig 2:**
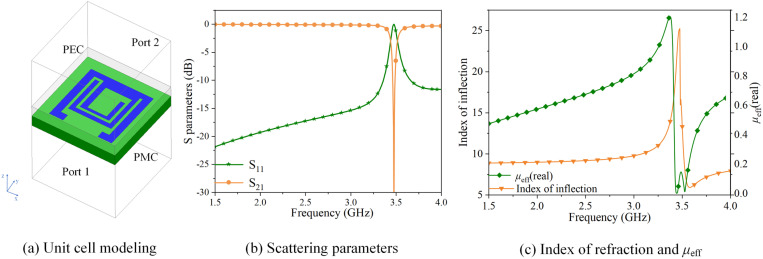
Toroidal metamaterials.

Electromagnetic metamaterials are composite materials engineered to achieve a tailored electromagnetic response through the artificial construction of periodic structures. The unique properties of these metamaterials enable their electromagnetic structures to interact with electromagnetic waves, leading to alterations in the propagation direction, phase, and other wave parameters. The model of the proposed metamaterials unit is proposed for miniaturized antenna decoupling, as shown in Fig 2.

Since the incident electromagnetic wave is *x*-polarized and propagates along the *y*-axis, we design the two surfaces parallel to the *xoz* plane as port 1 and port 2, the two surfaces parallel to the *xoy* plane as perfect electric conductors (PEC), and the two surfaces parallel to the *yoz* plane as perfect magnetic conductors (PMC). Fig 2 illustrates the electromagnetic characteristics of the toroidal metamaterials.

As depicted in Fig 2, the toroidal metamaterials exhibits resonance near 3.5 GHz, its negative permeability within the frequency range of 3.46 to 3.50 GHz, accompanied by a refractive index approaching 0. Consequently, the electromagnetic wave traversing the toroidal metamaterials behaves as a coupled current, which diminishes antenna coupling at 3.5 GHz. The waveguide layer is covered on the toroidal metamaterials units, which could further improve the performance of the wireless communication equipment and realize efficient decoupling and performance enhancement. By adding the proposed decoupling structure as Fig 1g shows, the MIMO mobile terminal antenna size is reduced by 49.43%.

### 2.3 Performance comparison of antenna before and after its miniaturization

**Fig 3:**
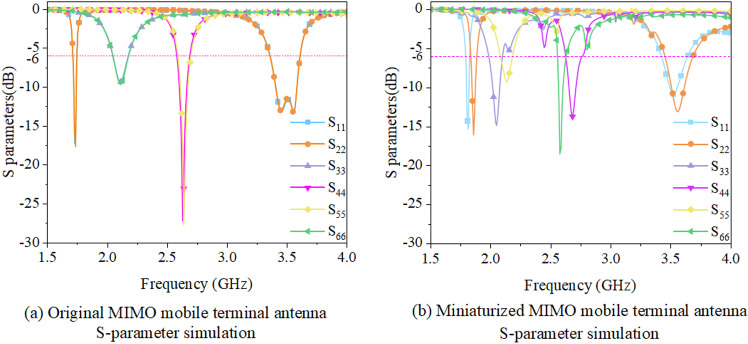
Comparison of S-parameter and gain simulation of antennas. (Unit: dBi).

In order to compare the radiation performance of the antennas before and after its miniaturization, S-parameter simulations are illustrated in Fig 3.

**Fig 4:**
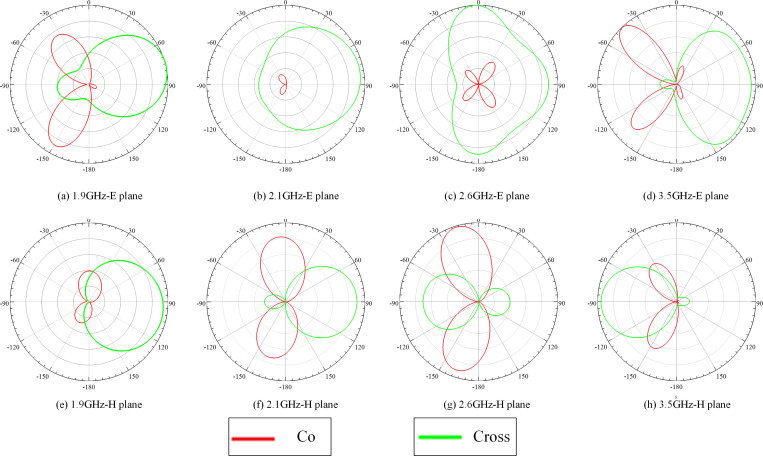
Radiation patterns at1.9 GHz, 2.1 GHz, 2.6 GHz, and 3.5 GHz.

Fig 3a, b demonstrate that the proposed miniaturized MIMO antenna for mobile terminals exhibits an *S*_11_ value of below -6 dB at frequency bandwidths of 1.875 ~ 1.925 GHz, 2.085 ~ 2.125 GHz, 2.55 ~ 2.64 GHz, and 3.41 ~ 3.55 GHz, which meet the MIMO mobile terminal antenna working on the current 2G, 3G, 4G, and 5G communication requirements. Furthermore, Fig 4 simulates the radiation direction diagram of the miniaturized MIMO mobile terminal antenna.

At 1.9 GHz, 2.1 GHz, 2.6 GHz, and 3.5 GHz frequencies, the simulated co-polarization and cross-polarization directions are shown in Fig 4. The co-polarization and cross-polarization patterns in these two planes are very acceptable for MIMO applications.

The gain simulations for the original MIMO mobile terminal antennas at 1.9 GHz, 2.1 GHz, 2.6 GHz, and 3.5 GHz are recorded as 2.78 dBi, 1.54 dBi, 2.55 dBi, and 4.92 dBi, respectively. In comparison, the corresponding gains for the miniaturized MIMO mobile terminal antenna are 1.3 dBi, 1.44 dBi, 2.23 dBi, and 4.11 dBi. The gain simulations indicate minimal variation in gain, suggesting that the proposed miniaturized antenna continues to fulfill the radiation performance requirements for MIMO mobile terminals.

### 2.4 Simulations and measurements comparison of miniaturized antenna

**Fig 5:**
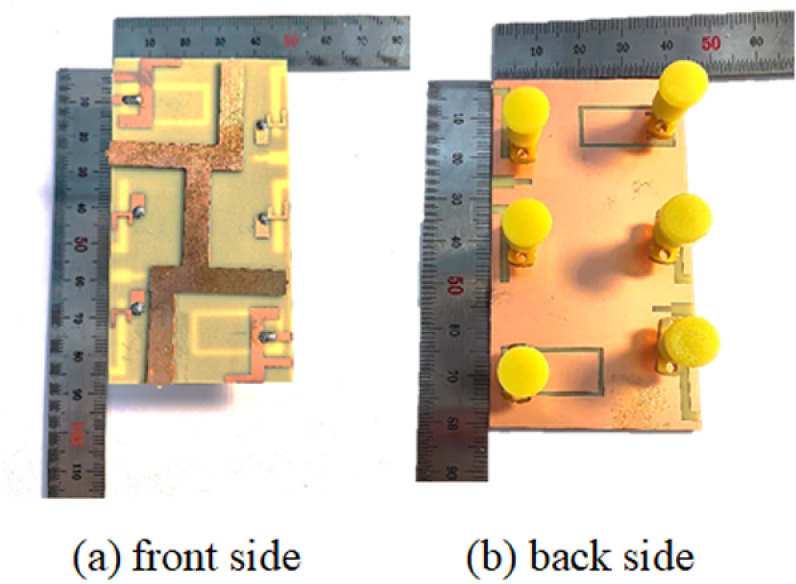
Fabrication of miniaturized MIMO mobile terminal antenna.

A prototype of the miniaturized MIMO mobile terminal antenna is fabricated, as shown in Fig 5a, b.

**Fig 6:**
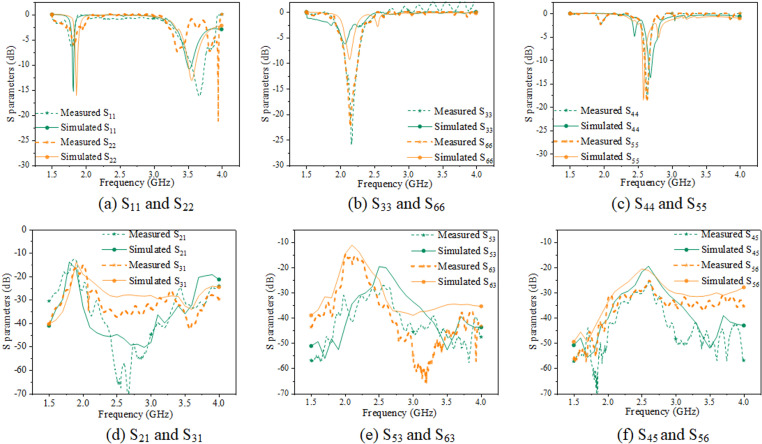
Simulated and measured S-parameters comparison of miniaturized MIMO mobile terminal antenna.

In order to enhance the validation of the proposed antenna’s reliability, its S-parameters are measured by Agilent Technologies vector network analyzer(VNA: SV4401A). Fig 6 presents a comparison between the simulated and measured S-parameters of the proposed antenna of S_11_, S_22_, S_33_, S_44_, S_55_, S_66_.

Fig 6 illustrates that the error between the simulations and measurements is acceptable, which is caused by the loss in the processing process. This finding demonstrates that the reliability of the proposed miniaturized antenna.

Table 1 compares the previously reported results with the proposed antenna structure in terms of overall antenna size, frequency band, isolation, efficiency, number of MIMO units and gain. It can be clearly observed from the table that compared with most reported structures, the proposed antenna has small overall size, fewer MIMO units but more frequency bands, high efficiency, high gain and low profile.

**Table 1:** Performance comparison of miniaturized mobile terminal MIMO antenna.

Ref	Board Size (mm^3^)	Frequency (GHz)	Isolation (dB)	Efficiency	MIMO Elements	Gain (dBi)
[6]	60 × 46 × 1.6	2.80-5.58	<-15	75.00% ~ 85.00%	4	2.46
[7]	136 × 68 × 0.8	3.3 ~ 3.7	<-15	50.00% ~ 75.00%	8	4
[14]	80 × 150 × 0.8	3.40 ~ 3.80 5.15 ~ 5.93	<-10	42.00% 62.00%	10	–
[36]	165 × 85 × 7	3.30 ~ 3.60 4.80 ~ 5.00	<-10	36.00% ~ 62.00% 46.00% ~ 63.00%	12	–
Proposed work	44 × 80 × 0.8	1.875 ~ 1.925 2.085 ~ 2.125 2.55 ~ 2.64 3.41 ~ 3.55	<-10	68.23% 88.08% 87.64% 96.23%	6	>3.20

## 3 Radiation effect of miniaturized MIMO mobile terminal antenna on human head tissues

### 3.1 Head modeling

**Fig 7:**
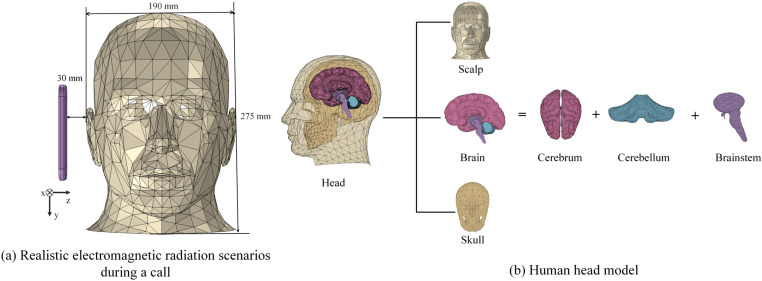
Human head modeling.

To better analyze the radiation doses absorbed by different tissues in the human head, a realistic representation of a human head is developed by using COMSOL software, adhering to Duke [37]. The dimensions of the constructed head model are 250 × 275 × 190 mm³, with a separation of 30 mm between the mobile terminal and the head model, as illustrated in Fig 7a.

According to epidemiological investigation, the susceptible important tissues to radiation inside the head are constructed based on medical images, as shown in Fig 7b.

The head model comprises five distinct tissue components: the cerebrum, cerebellum, brainstem, scalp, and skull. The relative permittivity and conductivity of these tissues at operating frequencies of 1.9 GHz, 2.1 GHz, 2.6 GHz, and 3.5 GHz are by the fourth Cole-Cole model [38,39], which was proposed by Gabriel in 1996, the corresponding dielectric parameters’ results are shown in Table 2.

**Table 2:** Human tissue dielectric parameters.

Frequency	Dielectric Property	Cerebrum	Cerebellum	Brainstem	Scalp	Skull
1.9 GHz	Relative permittivity	36.868	45.885	30.744	43.682	11.716
	Conductivity/Sm^-1^	0.957	1.765	0.878	1.283	0.292
2.1 GHz	Relative permittivity	36.60	45.462	30.514	43.365	11.592
	Conductivity/ Sm^-1^	1.046	1.882	0.951	1.390	0.328
2.6 GHz	Relative permittivity	35.991	44.544	29.997	42.645	11.293
	Conductivity/ Sm^-1^	1.292	2.201	1.151	1.684	0.424
3.5 GHz	Relative permittivity	35.003	43.143	29.173	41.473	10.793
	Conductivity/ Sm^-1^	1.810	2.865	1.574	2.308	0.615
	Tissue density/kg ∙ m^-3^	1038	1038	1038	1125	1990

### 3.2 Distribution comparison of radiation doses absorbed in different head tissues before and after antenna miniaturized

In order to elucidate the impact of radiation on the human head following the miniaturization of MIMO mobile terminal antennas, simulations are conducted to assess the radiation dose absorbed by various head tissues at operating frequencies of 1.9 GHz, 2.1 GHz, 2.6 GHz, and 3.5 GHz. The SAR is defined as the amount of electromagnetic radiation energy absorbed per unit mass of human tissue over a specified period. The formula for its calculation is presented in Equation (1):


$$SAR=\frac{\sigma {\left|E\right|}^{2}}{\rho }$$ (1)

A comparative analysis of MIMO mobile terminal antennas operating at a radiation power of 0.1W is conducted, focusing on the effects of antenna miniaturization. The variations in SAR distribution within the scalp, skull, cerebrum, cerebellum, and brainstem at various frequencies are simulated utilizing the FEM. The results of these simulations are illustrated in Figs 11–15.

**Fig 8:**
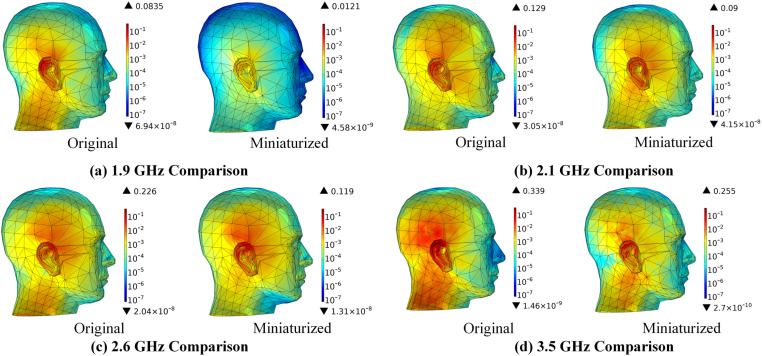
SAR distribution in the scalp comparison before and after antenna miniaturized (Unit: W/kg).

Fig 8 illustrates that, despite the continued increase in SAR in the scalp with rising frequency following the miniaturization of MIMO mobile terminal antennas, there is a notable reduction in both the distribution range of SAR and its corresponding values. Specifically, the maximum SAR values exhibit a decrease of 85.51% at 1.9 GHz, 30.23% at 2.1 GHz, 47.35% at 2.6 GHz, and 24.78% at 3.5 GHz. Furthermore, the SAR is predominantly concentrated in the area adjacent to the ear. According to reference [40], electromagnetic radiation has the potential to inflict ultrastructural damage on cochlear spiral ganglion cells under specific intensities and conditions. Consequently, the risk of ear exposure is significantly diminished following the miniaturization of antennas.

**Fig 9:**
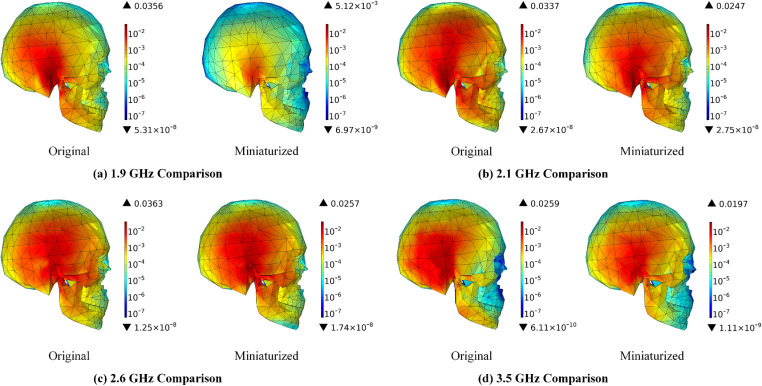
SAR distribution in the skull comparison before and after antenna miniaturized (Unit: W/kg).

Fig 9 illustrates that the variation in SAR within the skull is consistent with that observed in the scalp. The miniaturization of antennas is shown to significantly diminish the SAR distribution in the skull. Specifically, the maximum SAR values exhibit reductions of 85.62% at 1.9 GHz, 26.71% at 2.1 GHz, 29.20% at 2.6 GHz, and 23.94% at 3.5 GHz. An analysis of the SAR distribution across the skull reveals that the radiation energy is predominantly concentrated on the right side. Research [41] indicates that the influence of electromagnetic fields (EMF) on osteoblast differentiation is associated with EMF exposure. The miniature MIMO mobile terminal antenna proposed in this study effectively reduces the radiation dose absorbed by the skull, thereby potentially mitigating the risk of skeletal disorders in the cranial region.

**Fig 10:**
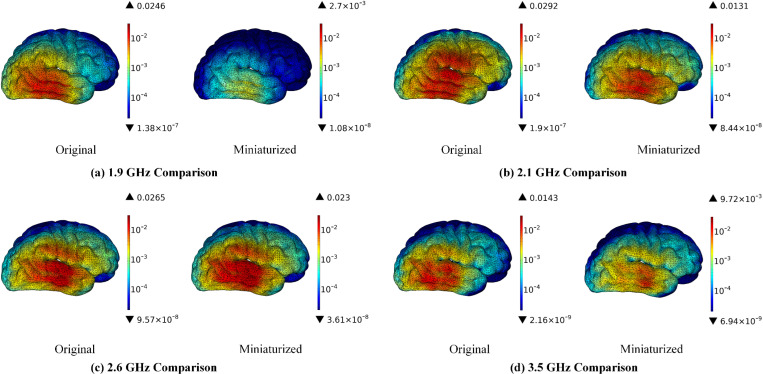
SAR distribution in the cerebrum comparison before and after antenna miniaturized (Unit: W/kg).

Fig 10 illustrates that the miniaturization of antennas can lead to a significant reduction in the SAR distribution within the cerebrum. The maximum SAR values exhibit a decrease of 89.02% at 1.9 GHz, 55.14% at 2.1 GHz, 13.21% at 2.6 GHz, and 32.03% at 3.5 GHz, respectively. An analysis of the SAR distribution in the cerebrum indicates that the radiation exposure is predominantly localized in the area directly opposite the antenna. Ref. [42] indicates that short-term acute exposure to radiofrequency electromagnetic radiation can result in the formation of carbon-centered lipid free radicals and damage to nuclear DNA, both of which may contribute to neurogenesis and neurodegenerative changes in the hippocampal region of the developing brain. Such effects may present as symptoms including headaches, dizziness, memory impairment, and difficulties with concentration. Consequently, this suggests that the proposed miniaturized antenna has the potential to mitigate the risk of adverse health effects on the cerebrum.

**Fig 11:**
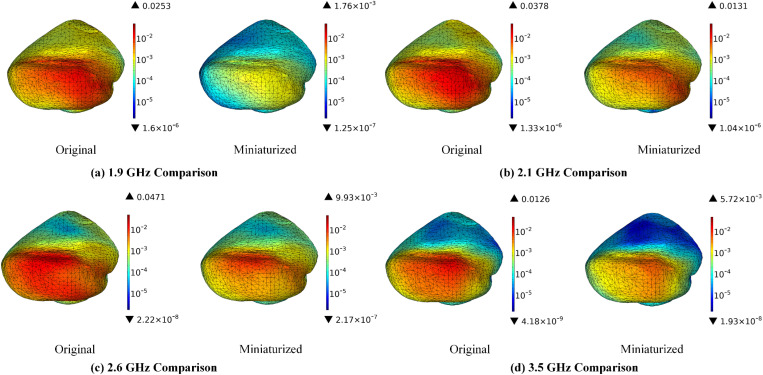
SAR distribution in the cerebellum comparison before and after antenna miniaturized (Unit: W/kg).

Fig 11 illustrates that the miniaturization of antennas can lead to a significant reduction in the SAR distribution within the cerebellum. The maximum SAR values exhibit decreases of 93.04% at 1.9 GHz, 65.34% at 2.1 GHz, 78.92% at 2.6 GHz, and 54.60% at 3.5 GHz, respectively. An analysis of the SAR distribution in the cerebellum indicates that the radiation exposure is predominantly localized in the area directly opposite the antenna. The cerebellum plays a crucial role in coordinating movement, maintaining postural balance, processing motor learning memories, and managing certain cognitive functions. Ref. [43] and [44] indicate that RF radiation may inhibit the activity of cerebellar protein kinases and adversely affect the histomorphological integrity of the cerebellum, as well as impair motor learning and memory capabilities. Consequently, the proposed miniaturized antennas may be beneficial in safeguarding the functional integrity of the cerebellum.

**Fig 12:**
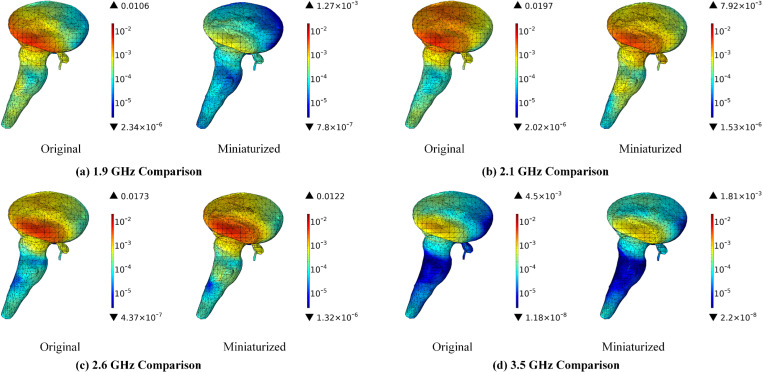
SAR distribution in the brainstem comparison before and after antenna miniaturized (Unit: W/kg).

Fig 12 illustrates that the miniaturization of antennas can lead to a significant reduction in the SAR distribution within the brainstem. The maximum SAR values exhibit a decrease of 88.02% at 1.9 GHz, 59.80% at 2.1 GHz, 29.48% at 2.6 GHz, and 59.78% at 3.5 GHz, respectively. An analysis of the SAR distribution in the brainstem reveals that the radiation is predominantly concentrated in the area directly opposite the antenna. The brainstem, which serves as a critical center for vital physiological functions such as respiration, cardiac activity, and consciousness, may be adversely affected by EMR, potentially leading to neurological dysfunction and endocrine disruption [45]. Therefore, the proposed miniaturized antennas may contribute to the preservation of the brainstem’s functionality and stability. Furthermore, it is indirectly corroborated that an increase in frequency corresponds to a decrease in wavelength and a reduction in penetration depth.

### 3.3 SAR variations for individual organizations due to frequency and radiation power effects

Due to the significant effect of frequency and input power on radiation dose, we further examine the influence of varying frequencies and radiation power on the MIMO terminal antenna. Initially, we compare the peak SAR values across different head tissues at various frequencies, both before and after the miniaturization of the antenna. The findings are presented in Table 3.

**Table 3:** Peak SAR values of different head tissues at different frequencies.

Frequency		Scale (W/kg)	Skull (W/kg)	Cerebrum (W/kg)	Cerebellum (W/kg)	Brainstem (W/kg)	ICNIRP limit (W/kg)
1.9GHz	Original antenna	0.0835	0.0356	0.0246	0.0253	0.0106	2
	Miniaturized antenna	0.0121	5.12 × 10^-3^	2.7 × 10^-3^	1.76 × 10^-3^	1.27 × 10^-3^	
	Diminution	85.51%	85.62%	89.02%	93.04%	88.02%	–
2.1GHz	Original antenna	0.129	0.0337	0.0292	0.0378	0.0197	2
	Miniaturized antenna	0.09	0.0247	0.0131	0.0131	7.92 × 10^-3^	
	Diminution	30.23%	26.71%	55.14%	65.34%	59.80%	–
2.6 GHz	Original antenna	0.226	0.0363	0.0265	0.0471	0.0173	2
	Miniaturized antenna	0.119	0.0257	0.023	9.93 × 10^-3^	0.0122	
	Diminution	47.35%	29.20%	13.21%	78.92%	29.48%	–
3.5 GHz	Original antenna	0.339	0.0259	0.0143	0.0126	4.50 × 10^-3^	2
	Miniaturized antenna	0.255	0.0197	9.72 × 10^-3^	5.72 × 10^-3^	1.81 × 10^-3^	
	Diminution	24.78%	23.94%	32.03%	54.60%	59.78%	–

The scalp, being in closest proximity to the antenna, is the primary site for the absorption of radiation energy emitted by the MIMO mobile terminal antenna. An increase in operating frequency correlates with a reduction in the peak SAR absorbed by the scalp, with a decrease of at least 24.78% observed following antenna miniaturization. At a frequency of 1.9 GHz, the peak SAR across various head tissues exhibits the most significant reduction, indicating that the miniaturized antenna effectively mitigates the penetration of lower frequency electromagnetic waves into human head tissues. The analysis presented in Table 2 demonstrates that the miniaturized MIMO terminal antenna significantly diminishes the risk of electromagnetic exposure to human head tissues associated with increased operational frequencies.

**Fig 13:**
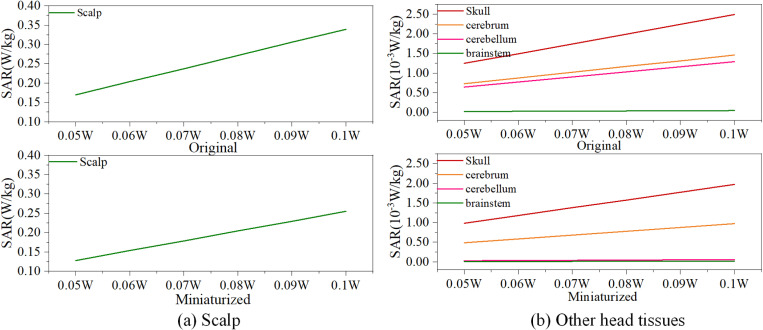
Variations in SAR for individual organizations.

Considering that the radiation power of mobile terminals can fluctuate depending on the communication environment, we further examine the variations in SAR under different radiation power levels before and after antenna miniaturization, as illustrated in Fig 13. Although the radiation dose absorbed by human tissues tends to increase with higher antenna radiation power, the miniaturization of the MIMO mobile terminal antenna results in a notable reduction in SAR within the scalp. Additionally, the rate of increase in SAR across various brain tissues, particularly in the brainstem, is correspondingly diminished. This evidence supports the conclusion that antenna miniaturization effectively curtails the escalation of radiation dose within the skull, thereby contributing to the protection of brain tissues from radiation exposure.

As indicated in Table 3 and Fig 13, the radiation exposure to cranial tissue can be mitigated through the miniaturization of the antenna, as well as by decreasing both the operating frequency and the radiation power.

## 4 Conclusions

This article presents a miniaturized MIMO antenna for mobile terminals and analyzes its effects on the human head. Key conclusions

(1) A MIMO mobile terminal antenna operating at 1.875 ~ 1.925 GHz, 2.085 ~ 2.125 GHz, 2.55 ~ 2.64 GHz, and 3.41 ~ 3.55 GHz is proposed. A novel decoupling unit is designed based on metamaterials units to realize the miniaturization of the proposed MIMO mobile terminal antenna, and its geometry is reduced by 49.43% without compromising radiation performance. Simulations and measurements confirm its suitability for 2G, 3G, 4G, and 5G communications.
(2) The miniaturized antenna significantly reduces SAR in head tissues compared to the original design. At 1.9 GHz, 2.1 GHz, 2.6 GHz, and 3.5 GHz, SAR values are reduced by 85.51% in the scalp, 85.62% in the skull, 89.02% in the cerebrum, 93.04% in the cerebellum, and 88.02% in the brainstem.
(3) The miniaturization of the antenna lowers peak scalp SAR by ≥ 24.78% at higher frequencies (e.g., 3.5 GHz), with the most reduction at 1.9 GHz. Besides that, the miniaturized antenna can effectively alleviate the trend of SAR increasing with input power, especially mitigating the SAR absorption in cranial tissues, limiting intracranial radiation influence, and protecting brain tissues effectively.

The conclusion reveals that the proposed miniaturized MIMO mobile terminal antenna could effectively reduce human tissue radiation absorption. It demonstrates that minimizing the geometry of radiation sources can lower radiation exposure, offering a novel approach for mobile terminal electromagnetic protection. Further research may advance safer, health-conscious wireless devices.
